# Novel graph-based centralized and decentralized approaches for early AKI prediction

**DOI:** 10.1038/s41598-025-31211-x

**Published:** 2025-12-06

**Authors:** V. S. Suresh Kumar, R. Devi Priya, M. Vijayakumar

**Affiliations:** 1 Department of Computer Science and Engineering, Nandha College of Technology, Erode, Tamil Nadu India; 2 Department of Computer Science and Engineering, KPR Institute of Engineering and Technology, Coimbatore, Tamil Nadu India; 3 Department of Computer Science and Engineering, Sasurie College of Engineering, Tiruppur, Tamil Nadu India

**Keywords:** Acute kidney injury, Graph attention network, Decentralized learning, Gossip learning, Physiological time-series, Predictive modeling, Data privacy, Clinical deployment, Biomarkers, Engineering, Materials science

## Abstract

Acute kidney injury (AKI) is a life-threatening problem for hospitalized patients, and early detection is crucial to reduce severe outcomes. Traditional predictive methods lack in monitoring complex physiological patterns and ensuring data privacy in decentralized healthcare settings. The study aims to develop and evaluate two distinctly complementary novel graph-based approaches, namely the centralized Graph Attention Network (GAT) and the decentralized model, Gossip Learning with Adaptive Aggregation GAT (GL-AA-GAT), to identify AKI onset between 6 and 12 h in advance, using physiological time-series data from the Kaggle Sepsis dataset. Multi-Head Attention is used for modeling feature interactions in centralized GAT, while GL-AA-GAT can further achieve this by decentralized training of five nodes using gossip exchange and adaptive aggregation for privacy and scalability. Through its novel graph structure, centralized GAT predicts the onset of AKI with an accuracy of 94.1%, sensitivity of 94%, AUC-ROC of 95%, and AUPRC of 91%. With decentralized privacy additions, GL-AA-GAT achieves an accuracy of 92.8%, a sensitivity of 93%, an AUC-ROC of 93.8%, and an AUPRC of 90%, with robustness. Sensitivity analyses revealed that the performance was stable with the prediction horizons and correlation thresholds, and external validation on non-sepsis cohorts of the ICU further indicated generalizability. Both models outperform existing models, which means high predictive reliability. The GL-AA-GAT’s distributed approach gives privacy and flexibility, making it innovative for distributed clinical environments, with task scheduling enhancing training efficiency.

## Introduction

AKI is an immediate loss of Renal activity, characterized by the accumulation of wastes and alterations in electrolytes and fluid overload^1^. The primary causes of AKI include dehydration, sepsis, heart surgery, nephrotoxic drugs, and other forms of critical illness like multiple organ failure. It increases the morbidity, length of hospital stays, and cardiovascular hazard: progressive CKD. A recent report indicated that in 2022, AKI affected 20.2% of hospitalized patients among Medicare Fee-for-Service (FFS) beneficiaries^2^. Early diagnosis and prompt intervention, such as dialysis, were considered highly crucial in clinical environments. Basically, AKI is diagnosed using the Renal Disease: updating Global Outcomes using KDIGO criteria^3^ and secondary biomarkers. In recent times, ML models and DL models have been implemented for pre-prognosis and severity prediction in AKI. These models are trained on patient vital signs, lab outcomes, and medication history, and they have demonstrated excellent predictive ability^4^. Similar high accuracy in AKI prediction has also been reported in deep learning models by identifying complex patterns^5^. These conventional table models do not address the relational dependencies defined within patient records, and are generally limited in terms of generalization and real clinical application. Moreover, scalability remains a major challenge, as many existing frameworks require heavy and critical computations, restricting their use in decentralized healthcare systems. The two independent approaches predict the early AKI in 6 to 12 h in advance. Centralized GATs learn complex interactions of features by creating patient-specific graphs from physiological time-series data and capturing complex dependencies between important clinical parameters such as creatinine, heart rate (HR), Temperature (Temp), BP [SBP, DBP, MAP], lactate, Resp, O2Sat, pH, WBC, and BUN that capture dependencies such as HR-creatinine, lactate-BP, and so on. GL-AA-GAT allows for the training of the model across five different nodes without exchanging raw data to extend the prediction of AKI into a privacy-preserving decentralized framework. Further, it employs task scheduling to optimize gossip exchange efficiency, thereby enhancing convergence efficiency with prediction. The Adaptive Aggregation (AA) mechanism ensures that only the most stable and accurate model updates from these nodes are added to the global GAT, making it a robust and scalable mechanism. The main contributions of the proposed work were:

1. The development of two independent novel graph-based models for the prediction of early AKI onset based on physiological features.
2. Multi-head attention in GAT allows this study to dynamically gather complex feature interactions between physiologic parameters like lactate-MAP, HR-creatinine, and so on.
3. Gossip learning in GL-AA-GAT provides privacy preservation to training without any exchange of raw data, which is ideal for multi-hospital settings.
4. The adaptive aggregation optimally decentralizes model updates by dynamically choosing accurate and stable contributions, thereby enhancing the process with improved robustness, scalability, and privacy.
5. The proposed two models performed better than existing models in both accuracy and area under the curve, with good calibration, which enables reliable early AKI prediction.
6. GL-AA-GAT improves scalability in decentralized settings by providing efficient convergence via task scheduling.

The study is organized as follows: “Literature review” explores the literature review and existing studies to identify research gaps. Section “Methodology” explains the proposed methodology. Section “Result and discussion” discusses the Model evaluation and performance metrics. Section “Conclusion” concludes with the main insights and future avenues of research.

## Literature review

AKI is a sudden dysfunction in the renal part. Early predictive information would make it possible to intervene timely manner and achieve better patient results. Traditional risk-scoring systems, namely, SOFA and SAPS II, demonstrate limited accuracy and generalization, which leads to the adoption of ML models^6^. The diverse ML algorithms, such as logistic regression (LR), k-nearest neighbors (KNN), support vector machine (SVM), decision tree (DT), random forest (RF), artificial neural networks (ANN), and extreme gradient boosting techniques (XGBoost), have been used for prediction. Among these, XGBoost scored 83% accuracy^7^, and, while the RF demonstrated 91% AUC^8^, both scored higher accuracy but lacked generalization. Feature selection methods such as recurring feature cancellation have been implemented for increased efficiency in model learning, with CatBoost^9^ and post-optimized LightGBM having shown higher AUC^10^. LASSO regression, obtaining an AUC of 79%, further adds interpretability by providing the most relevant predictors^11^. Feature elimination may reduce the prediction probability, which adversely affects the reliability of the Diagnosis.

On the other hand, ensemble models have been implemented with an AUC of 84%, maintaining robust performance^12^. Deep learning methodologies have improved AKI prediction, capturing temporal dependencies. An RNN-based model surpassed with an AUC of 0.893^13^. Meanwhile, a hybrid DL model using both patient records and ultrasound imaging achieved 95% AUC-ROC and 90% accuracy^14^. Bidirectional LSTM networks display strong predictive capabilities across all degrees of severity of AKI, with 95% AUC in prediction^15^. Deep learning techniques have also been employed in predicting serum creatinine variations^16^ and changes in urine output, scored with an AUC of 89%^17^. A multi-case prediction was achieved by an LSTM-reinforced RNN model that generated an RMSE value of 0.017 mg/dL^18^. Nonetheless, DL models suffer from overfitting issues and require high computational capabilities. To train a multi-institutional model^19^ without sharing the data directly, decentralized learning approaches such as federated learning (FL) have been proposed^20^. However, the integration of these approaches with graph-based learning is unexplored. Graph-based models have proven to be useful in capturing complex relationships in healthcare data and have shown promising outcomes in different applications. For instance, GATCDA obtained 0.9011 as an AUC value to achieve the prediction of circRNA-disease associations^21^, while iGATTLDA, which uses a transformer-based learning technique, reported 95% AUC for lncRNA-disease associations^22^.

Adaptive learning methods have also helped to improve feature fusion in gastrointestinal image classification^23^. Furthermore, ensemble learning and gossip learning (GL) have demonstrated high efficiency in decentralized environments. In cybersecurity, GL proved 98.82% accuracy in the detection of DoS attacks in V2X communication, demonstrating its promise for privacy-preserving, distributed model training^24^. A study^25^ developed a gossip learning framework to efficiently learn while scheduling tasks, thereby improving the convergence rate in a decentralized network, which acted as a fully distributed alternative to federated learning. In this context, a gossip-based SGD algorithm^26^ also allowed communication to take place during computation to speed up convergence in wide-area networks by using node exchanges as a priority. Similarly, GBTS^27^ targeted real-time tasks and dynamically gossiped about them over the first run, reducing communication, which is in a similar vein. Although these studies are suggestive of the potential, their application to AKI prediction is under-explored, a gap that is filled with accuracy-based task scheduling by the proposed GL-AA-GAT.

Despite these advancements, the existing models often face key challenges in AKI. Most of these models struggle to learn the variability of AKI in other clinical settings. The interpretability of the models is increased through the use of feature selection methods, but important predictors were discarded, which risks decreasing the reliability of the diagnosis. Overfitting and computation cost are the key challenges in DL, which make the real-time clinical setting challenging. FL is a decentralized training mechanism that ensures privacy preservation but does not exclude the existence of communication overheads, non-IID data distributions, and the lack of adaptive optimization of models. The previous studies^6–18^ have rarely used the graph-based learning model, which can learn complex interdependencies among clinical parameters like lactate-MAP, creatinine-SBP, and so on. Integrating graph-based learning with decentralized and adaptive aggregation, along with task scheduling for efficient training, remains a critical research gap. To address these challenges, this study proposed two independent approaches: GAT and GL-AA-GAT. The GAT effectively improves interpretability and accuracy for early AKI prediction. It handles irregular time-series data and scales up for deployment in a centralized way. The GL-AA-GAT allows secure decentralized training over multiple nodes, without the necessity of exchanging raw data. It ensures scalability and robustness with a high accuracy rate.

The recent advancements have emphasized the effectiveness of hierarchical and dynamic attention in biomedical graph modeling. As an example, a study^28^ has HDGAT combining hierarchical and dynamic attention to improve drug-disease alliance prediction, which previous models lacked interpretability or robustness in many cases. Generation AI to generate synthetic health data is also an indicator that privacy-preserving analysis can be implemented, with^29^ reporting the solution to the problem of clinical data sharing and open provision of EHR. Further, the hybrid representation learning with Transformer and GNN architecture in multi-task decision-making applied to CKD management, as shown by^30^, achieves the state-of-the-art results in both progression and intervention recommendation, which is a strong argument in favor of the benefit of this method in nephrology. Along with these progresses, this paper introduces a graph-based centralized and decentralized early AKI prediction model, aiming to optimize clinical accuracy and privacy, while maintaining a scalable design in the future.

## Methodology

**Fig. 1:**
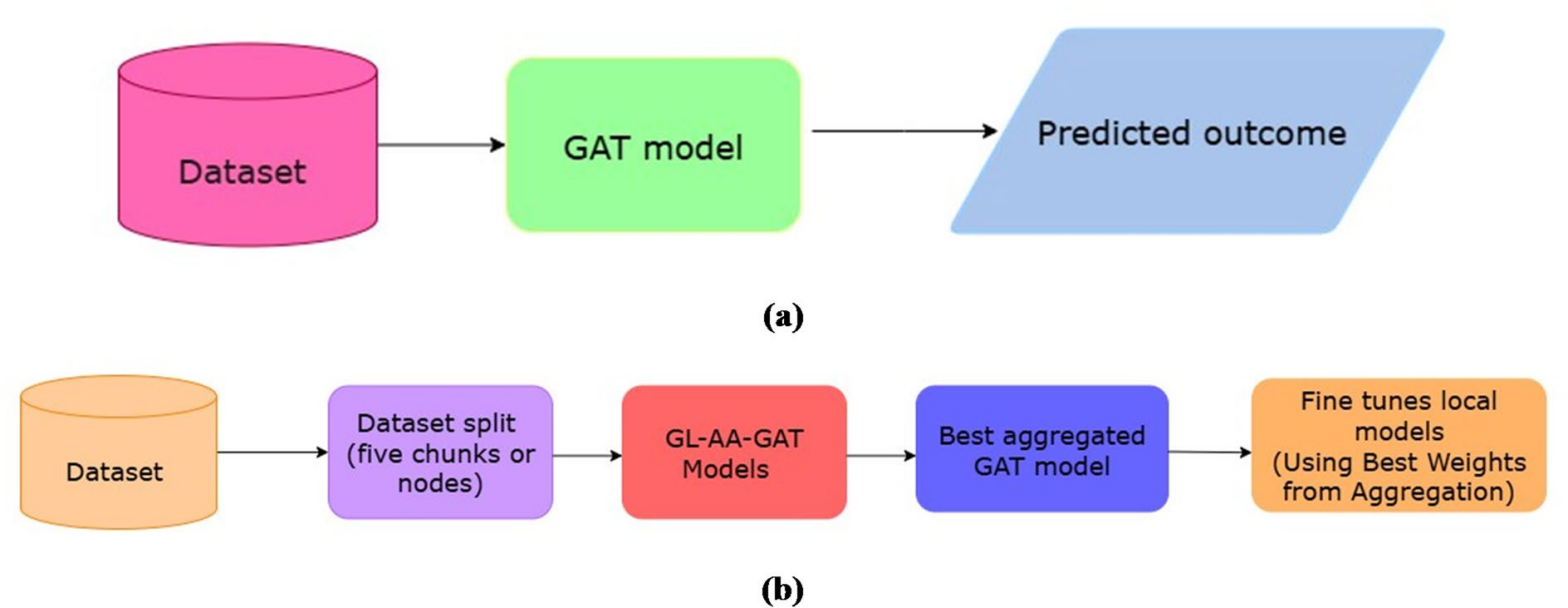
The proposed two independent approaches architecture diagrams. (**a**) Overall Architecture of GAT approach. (**b**) Overall architecture of GL-AA-GAT aproach.

The flow of the proposed two independent approaches is displayed in Fig. 1a, b. In the GAT approach, by transforming physiological time-series data, the model generates patient-specific graphs that preserve complex feature interactions such as creatinine-BP-BUN-BUN, which are key indicators for early AKI detection.

A decentralized GL-AA-GAT approach in the form of random peer-to-peer exchanges, followed by adaptive aggregation, adds the capability of increasing privacy and scalability as perceived by the approach.

### Dataset and preprocessing

The Kaggle Sepsis dataset^31^, employed in this research, is publicly accessible and fully de-identified, which guarantees adherence to ethical principles of data use. It consists of 44 attributes and 40,336 patient records, with hourly data including creatinine (mg/dL), SBP, DBP, MAP (mmHg), HR, Temp, Resp, Lactate, O2Sat, BUN (mg/dL), pH, and WBC. These features are chosen on the grounds of significant clinical relevance to AKI progress and sepsis dynamics, thereby embedding vital key physiological signals for graph-based modeling. The duration of the patient’s records ranges from 1 to 336 h, averaging about 24 h with a standard deviation of 10 h. The AKI events are identified according to the KDIGO criterion, which states that AKI is diagnosed when an increase of creatinine by 0.3 mg/dL occurs within 48 h. The first inpatient value was baseline creatinine; when this value was unavailable, we reconstructed it using the MDRD (estimated Glomerular Filtration Rate) equation: eGFR = 75 mL/min/1.73m^2^. A patient is considered AKI-positive when creatinine rose by $\:\ge\:$ 0.3 mg/dL in 48 h or $\:\ge\:$ 1.5 times baseline in 7 days. Records without creatinine were excluded.

The 48-hour prediction horizon was thus used to align with the KDIGO guidelines^32^ to achieve clinical unity in outcome labeling. This window has been confirmed in numerous previous studies as stable and highly predictive of AKI^13^. compared both 24 h and 48 h horizons and found that the discriminative ability was reliable at 48 h, whereas^33^ established that the comparability of results is stable across horizons as defined by KDIGO. Another source that supported the use of KDIGO 48 h definitions in practice was the multicenter modeling by^5,34^. Another recent systematic review also pointed out 48 h as the most frequent and clinically significant point of AKI modeling^35^. This evidence supports the 48 h window in our framework, both clinically and methodologically. The data are prepared by preprocessing each patient’s record to create a time-windowed graph for the 48 h preceding AKI onset. Normalization of the features via z-scores constitutes an additional step for maintaining uniformity across records for proper approach training.

### Data split

The data is partitioned for centralized and decentralized experiments while ensuring statistical reliability. For a centralized GAT operation, the split used is 85:15 to balance training data availability with robust testing, which means 34,286 records are trained and 6050 records are held out for testing. On the other hand, GL-AA-GAT is tested on 6050 records, while training on 34,285 records is split into five equally sized subsets of 6,857 records each, simulating decentralized learning across five nodes. All splits consistently maintain an AKI prevalence of 15%, which mirrors the real-world incidence rates and ensures balanced class representation. All splits consistently have a 15% AKI prevalence, as in the real-world incidence rates, indicating balanced class representation. Among all splits, the most renowned by machine-learning practice is 85:15, giving the subsequent training set access to sufficient data while excluding enough for a test set for evaluation. This five-fold partitioning has GL-AA-GAT mimic federated learning situations in the healthcare environment, thus increasing scalability and upholding privacy.

### Graph construction

Using selected clinical biomarkers for creatinine, blood pressure, and BUN, a patient-specific graph is built to represent the interdependencies among physiological characteristics: these would be nodes in our graph. The weights on edges between these nodes are designated based on temporal correlations among the features, thus preserving only clinically meaningful connections. The edge weight between the two features $\:{x}_{i}$ and $\:{x}_{j}$ at time $\:t\:$computed as follows by the Pearson correlation coefficient:

1 $$\:{w}_{ij}=\left\{\genfrac{}{}{0pt}{}{Pearson\left({x}_{i},{x}_{j}\right)}{0}\:\genfrac{}{}{0pt}{}{\:\:\:\:\:\:\:\:\:\:\:\:\:\:\:\:\:\:\:\:\:\:\:\:\:\:\:if\left|Pearson\left({x}_{i},{x}_{j}\right)\right|>0.3,}{otherwise,}\right.$$

Here, $\:\:{w}_{ij}$ indicates the strength of the connection between two physiological features. Edges between features were constructed using Pearson correlation, and the threshold of 0.3 was chosen to maintain clinically significant associations and eliminate weak or spurious ones. Statistical guidance classifies |r| $\:\ge\:$ 0.3 as the lower bound of weak correlations that are interpretable when applied to biomedical studies^36^. This kind of threshold (0.25–0.35) has been broadly used in biomedical construction of graphs in order to trade interpretability against noise reduction^37^. Empirical studies on AKI prediction demonstrated that the stability of models is maintained when the threshold is changed slightly in this range^33^. Therefore, *r* = 0.3 is both statistically and empirically sound. This structure of the graph is subject to dynamic temporal evolution, which captures changes in physiological responses that can signify the onset of AKI. $\:{x}_{i}$ and $\:{x}_{j}\:$are the time-series vectors associated with features $\:i$ and $\:j$. Each node within this graph is initialized with a feature vector capturing its evolution in time over the past three steps only, not utilizing any information from the 48-hour history. The feature vector for a node corresponding to physiological feature$\:\:i$ at time $\:t$ is computed as follows:

2 $$\:{h}_{i,t}^{\left(0\right)}=\left[{x}_{i,t},{x}_{i,t-1},{x}_{i,t-2}\right]$$

where $\:{h}_{i,t}^{\left(0\right)}$ denotes the initial state of the node, which consists of the most recent measurement$\:{\:x}_{i,t}$ in conjunction with values taken from the measurements two time steps back,$\:\:{x}_{i,t-1}$ and $\:{x}_{i,t-2}$. This temporal representation is suitable since AKI is an acute event that is less about long-term trends and more about rapid physiologic change. A history of 48 h would rather add some noise and complexity without providing a meaningful boost in predictive power.

#### Centralized graph attention network (GAT) implementation

**Fig. 2:**
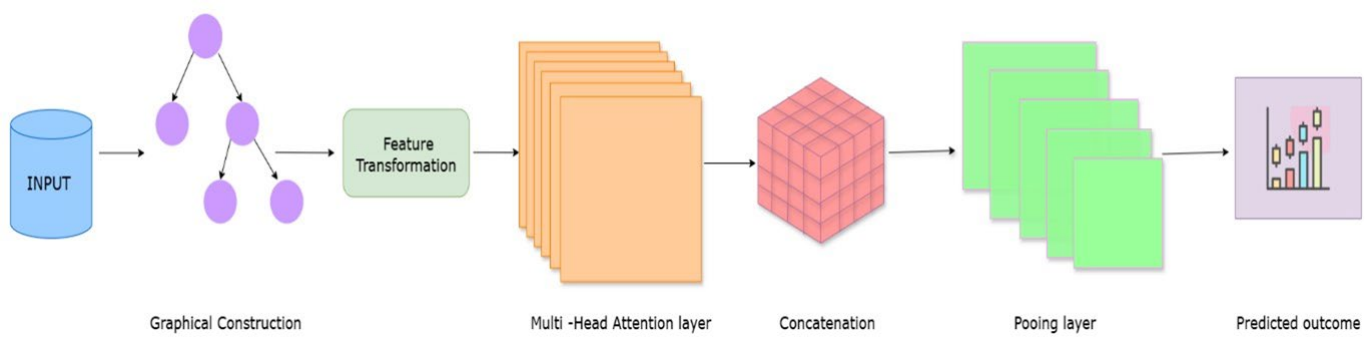
Centralized GAT.

This GAT models the propagation of information across the physiological feature graph, as presented in Fig. 2. Each node collects information from its neighbors based on learned attention scores. Before the application of attention mechanisms, the node features are transformed into a higher-dimensional latent space to enhance the expressiveness of feature interactions:

3 $$\:{h}_{i,t}^{\left(1\right)}={W}^{\left(1\right)}{h}_{i,t}^{\left(0\right)}$$

where, $\:{h}_{i,t}^{\left(1\right)}$ is the transformed feature vector of size $\:{d}_{h}$=64 and is a trainable weight matrix of shape $\:{\mathbb{R}}^{{d}_{h}\times\:{d}_{0}}$, where $\:{d}_{0}=3$ as represented in 3-dimensional input features to the 64-dimensional hidden space $\:{d}_{h}=64$. This helps the model capture non-linear relationships between different physiological features to help improve early sign detection of AKI. The main component of a GAT model is this multi-head attention, which assigns different importance weights to different physiological interactions. For each node pair $\:i$ and $\:\:j$, the attention is computed in an unnormalized form as:

4 $$\:{e}_{ij}^{k}=LeakyReLU\left({a}^{k}.\left[\left.{W}^{k}{h}_{i,t}^{\left(1\right)}\right|\left|{W}^{k}{h}_{j,t}^{\left(1\right)}\right.\right]\right)$$

where,$\:{\:e}_{ij}^{k}$ represents the relevance of node $\:i$ and $\:j$ in the attention score for head k, $\:{a}^{k}$ is a learnable attention vector of size $\:{\mathbb{R}}^{{2d}_{h}}$, where $\:{2d}_{h}=128$ due to concatenation $\:{W}^{k}$ is the transformation matrix for attention head$\:\:k$, and $\:\parallel\:$ denotes the concatenation. The normalized attention coefficient defines how much information node i should feed onto node j; hence, it was computed via the softmax function:

5 $$\:{\alpha\:}_{ij}^{k}=\frac{exp\left({e}_{ij}^{k}\right)}{{{\Sigma\:}}_{m\in\:{\mathcal{N}}_{i}}exp\left({e}_{im}^{k}\right)}$$

where, $\:{\alpha\:}_{ij}^{k}$ stands for the normalized attention weight assigned to node $\:j$ while aggregating information for node $\:i$, and $\:{\mathcal{N}}_{i}$ is the set of nodes that are in direct connection with node i in the graph. The attention score for neighbor m is given as $\:{e}_{im}^{k}$. The updated feature representation for node i is then computed as:

6 $$\:{h}_{i,t}^{\left(k\right){\prime\:}}=\sigma\:\left(\sum\:_{j\in\:{\mathcal{N}}_{i}}{\alpha\:}_{ij}^{k}{W}^{k}{h}_{j,t}^{\left(1\right)}\right)$$

σ is the ReLU activation function. This entire process is iterated over K = 8 independent attention heads, and the output is concatenated to form the final multi-head representation:

7 $$\:{h}_{i,t}^{\left(2\right)}={concat}_{k=1}^{K}{h}_{i,t}^{\left(k\right){\prime\:}}$$

resulting in a feature dimension of $\:8\times\:64=512,\:$where$\:{\:\:d}_{h}=64$ is the hidden dimension and $\:K=8$ denotes the number of attention heads. The attention mechanism allows each head to capture distinct physiological dependencies and dynamically prioritize those feature interactions within the 48-hour window. Setting $\:{d}_{h}=64$ makes each head responsible for discovering specific interaction patterns, for instance, the creatinine-SBP relationship caused by perfusion-related issues, and together with eight heads, it covers all the physiological dynamics. The resulting feature vector $\:{h}_{i,t}^{\left(2\right)}$ delivers multiple interaction perspective integration, consistent with the need to detect subtle onset signals. The second GAT layer is used to further refine the learned representations and hence, the final node $\:{h}_{i,t}^{\left(3\right)}$ embedding is created, which acts as input for global feature aggregation. Node embeddings are being pooled across feature and time dimensions to derive a comprehensive patient-level representation.

8 $$\:{h}_{global}=\frac{1}{NT}\sum\:_{i=1}^{N}\sum\:_{t=1}^{T}{h}_{i,t}^{\left(3\right)}$$

where, $\:{h}_{global\:}$stands for global feature vector, $\:N$ is the number of physiological features (nodes), and $\:T$ is the time steps. Node embedding captures interactions of multiple time-step features; this aggregation provides a compact yet highly informative summary of the physiological state of the patient. Eventually, a fully connected layer leading to a sigmoid activation function is used to predict the probability of developing AKI in the next 6 to 12 h:

9 $$\:{P}_{AKI}=sigmoid\left({W}_{fc}{h}_{global}+b\right)$$

$\:{P}_{AKI}$ signifies the predicted probability of occurrence of AKI. The probability is derived using a sigmoid activation function on a weighted summation of the global representation of features, which comprises key clinical attributes derived from patients’ data. The weight matrix of the fully-connected layer (FC)$\:\:{W}_{fc}$​ acts on these features, and the bias term $\:b$ alters the output. The sigmoid activation ensures the output lies inside [0,1] so that it can be interpreted as a probability. Global pooling integrates the feature-first and temporal signals before the fully connected layer and increases model sensitivity in detecting the dispersed-onset patterns of AKI. The model is trained by Adam optimizer (learning rate = 0.001), cross-entropy loss, and 50 epochs, and attention weights are refined for centralized data. The training was done on an NVIDIA GPU server (equivalent performance achievable with current CPU clusters), and the inference runs near real time, making it viable to deploy to hospitals at scale.

#### GL-AA-GAT with global model distribution and fine-tuning

**Fig. 3:**
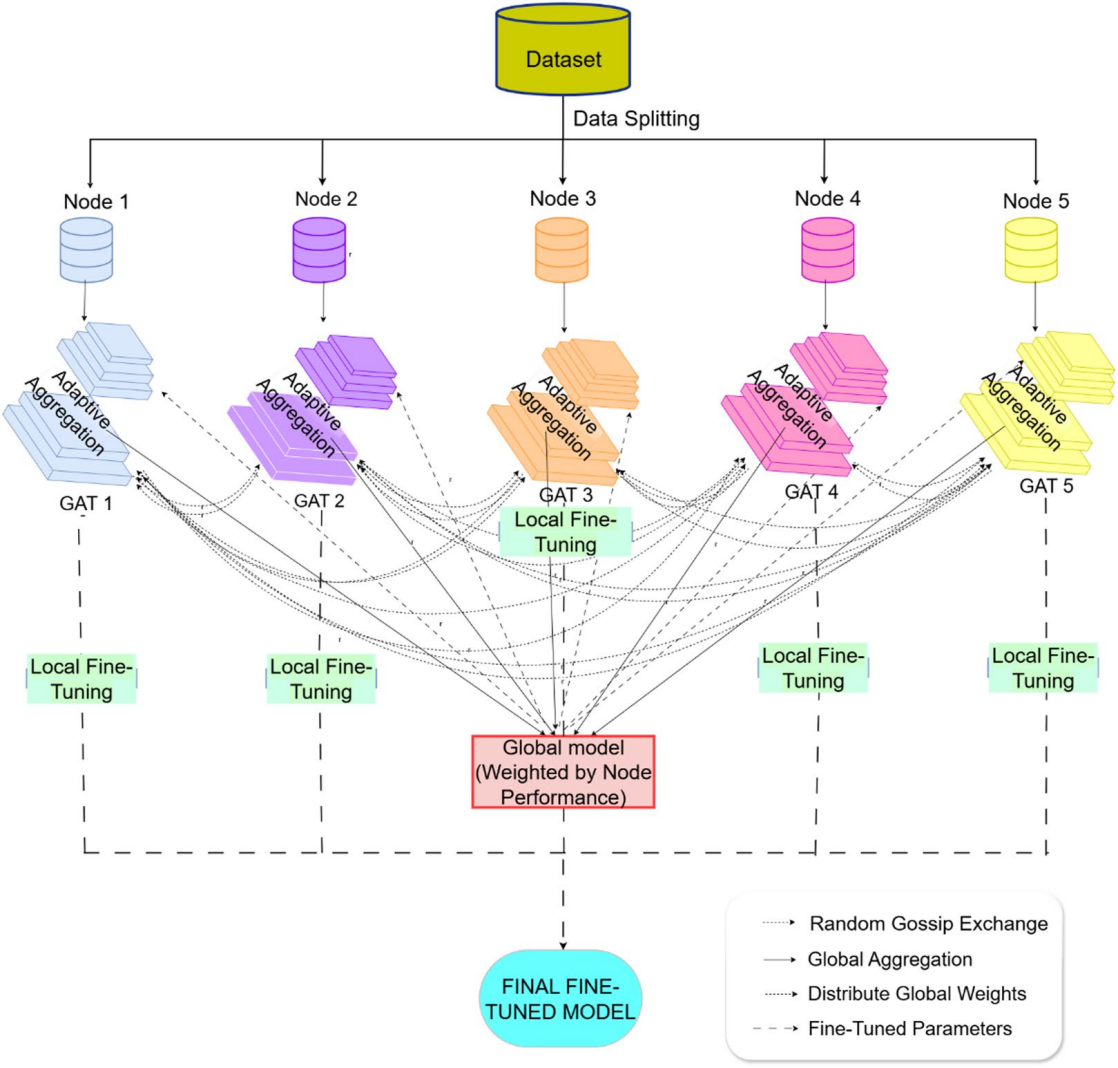
GL-AA-GAT architecture.

The GL-AA-GAT architecture is displayed in Fig. 3. The model trains GAT models across five nodes, aggregates them to a global model, and then distributes the global model to local fine-tune for better privacy and adaptability. Each node trains a local GAT on its subset using the Centralized GAT architecture (Eqs. 3–7).

For local training, each node trains a GAT model on its local dataset of 6857 records using the same architecture as Centralized GAT. Thus, each node captures the patterns specific to its data, for example, the hospital-specific trends in creatinine. The gossip exchange mechanism empowers nodes to engage in exchanging parameter updates with a randomly chosen peer after every epoch.

10 $$\:{\varDelta\:\theta\:}_{n}={\theta\:}_{n}^{\left(t\right)}-{\theta\:}_{n}^{(t-1)}$$

For node n, the dimensionality equals the model parameters. Here, $\:{\theta\:}_{n}^{\left(t\right)}$ and $\:{\theta\:}_{n}^{(t-1)}$are the local parameters of node $\:n$ at epoch $\:t$ and $\:t-1,$ respectively, and $\:{\varDelta\:\theta\:}_{n}$ is the parameter update that signifies the change in weights. The decentralized exchange design delivers scalability together with privacy because it restricts communication to one single peer during each round without storing centralized data. The adaptive aggregation step builds a global model by summing weighted local models that rely on validation accuracy for their respective weights.

11 $$\:{\theta\:}_{global}=\sum\:_{n=1}^{5}{p}_{n}{\theta\:}_{n}$$

where, $\:{\theta\:}_{global}$ global model parameters, dimension matching $\:{\theta\:}_{n}$ which is a local parameter of node $\:n$. $\:{p}_{n}$ represents the weight for node $\:n$. Weights are calculated as,

12 $$\:{p}_{n}=\frac{exp\left({acc}_{n}\right)}{\sum\:_{m=1}^{5}exp\left({acc}_{n}\right)}$$

where$\:\:{p}_{n}$ is the normalized weight, scalar in [0, 1], summing to 1 across nodes. $\:{acc}_{n}$ is the validation accuracy of the local model of node n. In the adaptive aggregation, a global model is synthesized by weighting local contributions according to their accuracy, weighting those nodes that showed better AKI detection. The aggregated global model is distributed to all five nodes:

13 $$\:{\theta\:}_{n}^{\left({t}_{agg}\right)}={\theta\:}_{global}$$

where, $\:{\theta\:}_{n}^{\left({t}_{agg}\right)}$ is the parameters of node n, which is initialized with the global model $\:{\theta\:}_{global}$, at the time of aggregation $\:{t}_{agg}$. Finally, the local fine-tuning makes the global model fit the local data of each node:

14 $$\:{\theta\:}_{n}^{\left({t}_{fine}\right)}={\theta\:}_{n}^{\left({t}_{agg}\right)}-\eta\:\nabla\:L\left({\theta\:}_{n}^{\left({t}_{agg}\right)},{D}_{n}\right)$$

The update step is controlled by the learning rate $\:\eta\:=0.0001$, $\:{D}_{n}$ is the local dataset of node $\:n$, and $\:\nabla\:L\left({\theta\:}_{n}^{\left({t}_{agg}\right)},{D}_{n}\right)$ is the gradient of the cross-entropy loss concerning the global model parameters computed on $\:{D}_{n}$. $\:{\theta\:}_{n}^{\left({t}_{fine}\right)}$ is the parameter set after a few epochs, with global knowledge and local specificity balanced. This step also adheres to the principles of transfer learning, where a robust baseline can be adapted to different healthcare environments. The algorithmic process of both approaches is provided in Algorithms 1 and 2.

**Figure:**
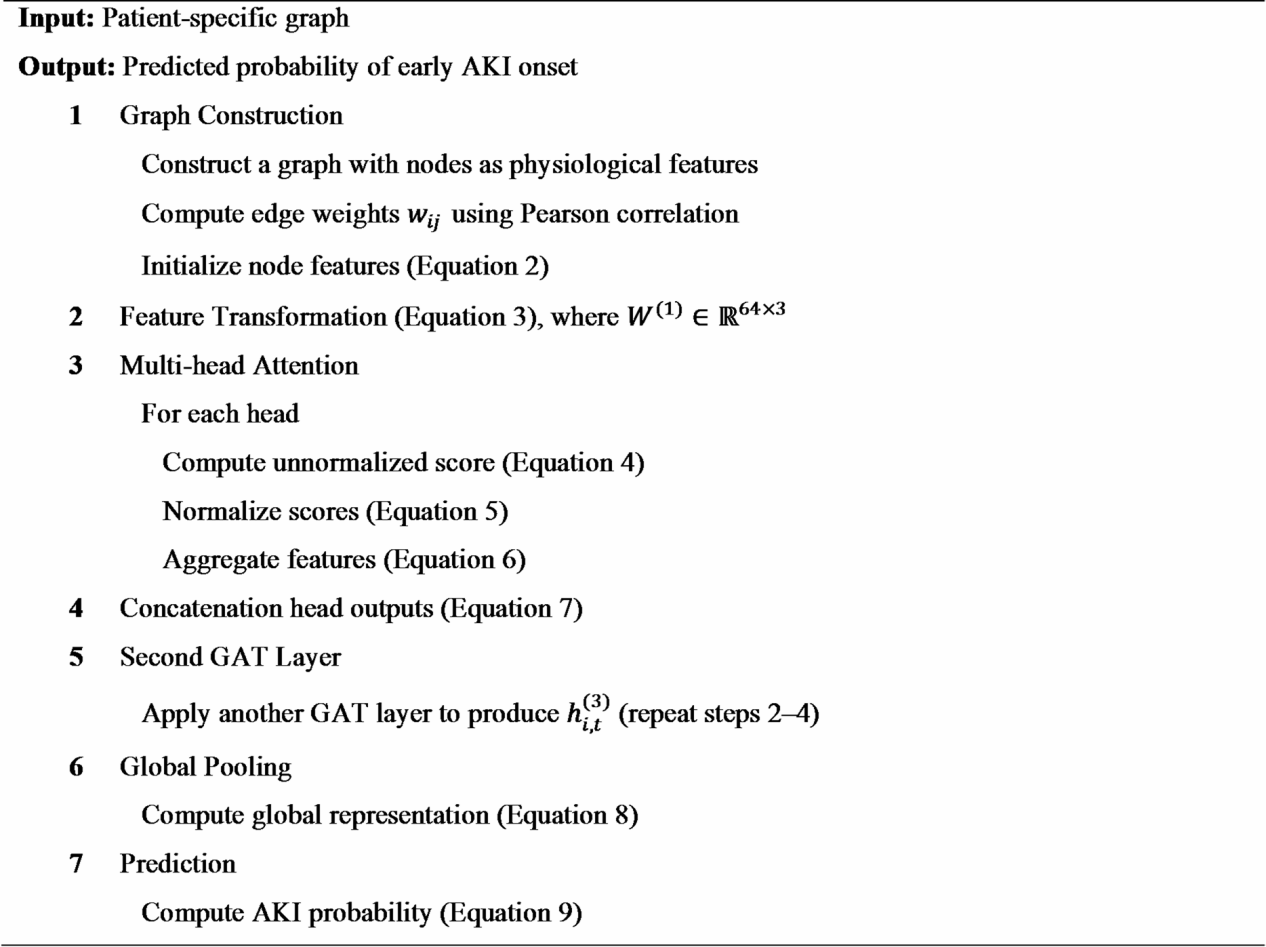
**Algorithm 1** Centralized GAT for early AKI prediction.

**Figure:**
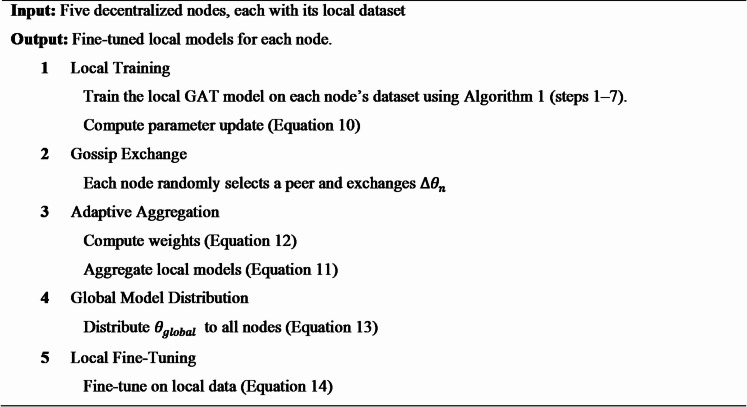
**Algorithm 2** GL-AA-GAT for early AKI prediction.

The time cost of algorithm 1 (centralized GAT training) is $\:O(E\cdot d\cdot H)$ per layer, as $\:E$ denotes the count of edges, $\:d$ represents the dimension of the feature, and $\:H$ is the count of attention heads. That cuts about 7.2k $\:\times\:$ per improvements in the case of the 30variables/graph, d = 3-time steps, and H = 8, i.e., is not very significant to the capability of GPUs. GL-AA-GAT gossip updates (Algorithm 2) contain $\:O\:\left(P\right)$ communication per round, $\:P$ is the number of parameters, and this is approximately 27 MB. Gossip reduces about 27 MB of communication a round compared to approximately 135 MB of communication a round in FL ($\:O(N\bullet\:P)$ a round ($\:N$ nodes)). The gossip causes a reduction of more than 4 times in the 5-node system.

For the Centralized GAT approach, this study employed eight attention heads, which were optimized by 5-fold cross-validation to improve the feature interaction modeling. Hyperparameters were tuned via grid search: $\:lr\:\in\:\:\{{1e}^{-4},{5e}^{-4},{1e}^{-3}\}\:\to\:\:{1e}^{-3};\:heads\:\in\:\:\{\text{1,4},\text{8,12}\}$; hidden size $\:\in\:\:\left\{\text{32,64,128}\right\}$; dropout $\:\in\:\:\left\{\text{0.2,0.3,0.5}\right\}$; batch $\:\in\:\:\left\{\text{64,128,256}\right\}$ with a atmost of 200 epochs and early stopping. The learning rate was set to 0.001, there were eight heads, a hidden size of 64, a dropout value of 0.3, and the batch size was 128. This was chosen such that diverse physiological patterns can be captured with sufficient computational efficiency, with alternative configurations such as 1, 4, and 12 heads assessed to confirm optimality. A 48-hour window was selected to correlate with KDIGO’s AKI definition^3^ and acute physiological changes, as validated in^15^. Edge weights were calculated using Pearson correlation with a 0.3 threshold, which was selected for ease of computation and clinical connection in capturing linear relationships^10^. For the GL-AA-GAT approach, 18 gossip rounds were determined according to the convergence behavior among five decentralized nodes, whereas task scheduling was used to further assess and enhance the running efficiency by pinpointing accuracy updates. To stabilize model updates with its contribution, Adaptive aggregation was applied and assessed in the Results. To benchmark, the two common ICU scoring systems were also used in this study: SOFA and SAPS II. Since the data lacked Glasgow Coma Scale, urine output, and comorbidity variables, we applied modified SOFA and partial SAPS II scores. Specifically, the PaO_2_/FiO_2_ ratio was estimated by means of available FiO_2_ and O_2_ saturation, and items concerning chronic diseases were omitted. These partial applications are in line with the secondary findings in ICU AKI cohorts^38,39^. Detailed performance outcomes for both approaches are reported in the Results and Discussion section.

#### Task scheduling for efficient gossip exchange

In the GL-AA-GAT model, Task Scheduling adjusts the gossip exchange process by prioritizing those nodes that have higher local validation accuracy during the process of parameter updates. Instead of random peer selection, which is the default mechanism across 18 rounds, nodes are sorted according to their accuracy, and exchanges are scheduled such that updates of the first best nodes are shared first, thus requiring less time to reach convergence while still preserving the predictive accuracy. The algorithmic process of task scheduling in the GL-AA-GAT approach is provided in Algorithm 3.

**Figure:**
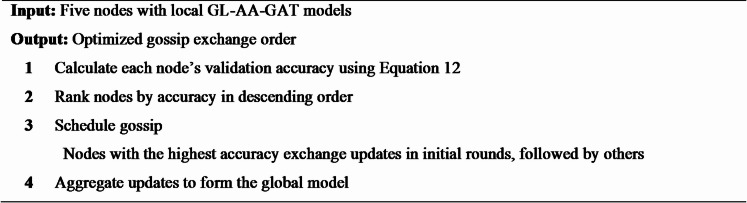
**Algorithm 3** Task scheduling for gossip exchange.

This ranks the nodes using the accuracy while scheduling the gossip exchanges to propagate only retentive updates, thus revamping the training way quicker; therefore, it facilitates scalability for potential multi-institutional deployment with no loss of privacy and robustness.

#### Experimental setup

The centralized GAT approach was trained with 32,269 patient graphs from the dataset^31^ in 20 min on an NVIDIA RTX 3080 GPU, and the inference time was 5ms, making it possible to do real-time AKI prediction in centralized healthcare systems. The GL-AA-GAT approach required 25 min per node, with an inference time of 6ms, which enables the real-time prediction of decentralized settings.

## Result and discussion

The two independent models utilize graph-based learning to learn physiological relationships, and GL-AA-GAT takes this a step further by making it capable of learning in a decentralized environment with privacy preservation. Centralized GAT and GL-AA-GAT are evaluated in terms of multiple evaluation metrics to fully understand their clinical utility.

### Centralized GAT performance evaluation

**Fig. 4:**
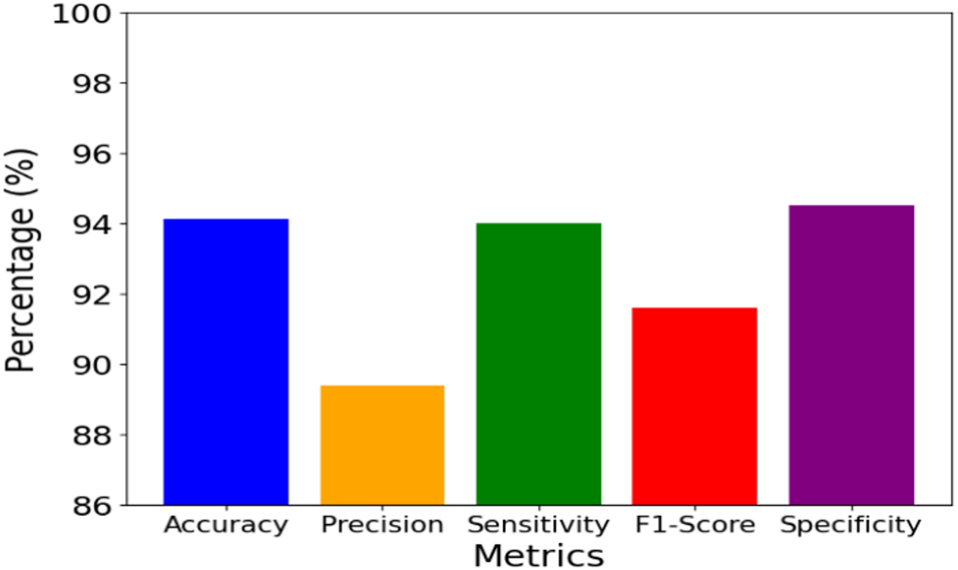
GAT model performance metrics for early AKI prediction.

The performance metrics of the centralized GAT approach are illustrated in Fig. 4.

**Fig. 5:**
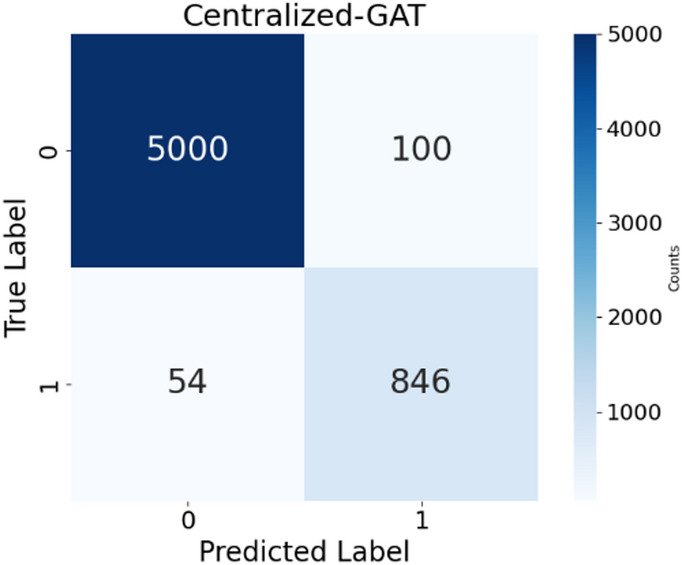
GAT model confusion matrix.

This study achieves 94.1% accuracy in centralized GAT on the prediction of AKI 6–12 h before onset based on its graph-based attention mechanisms. The confusion matrix shown in Fig. 5 highlights the classification performance of the GAT model in predicting early AKI.

**Fig. 6:**
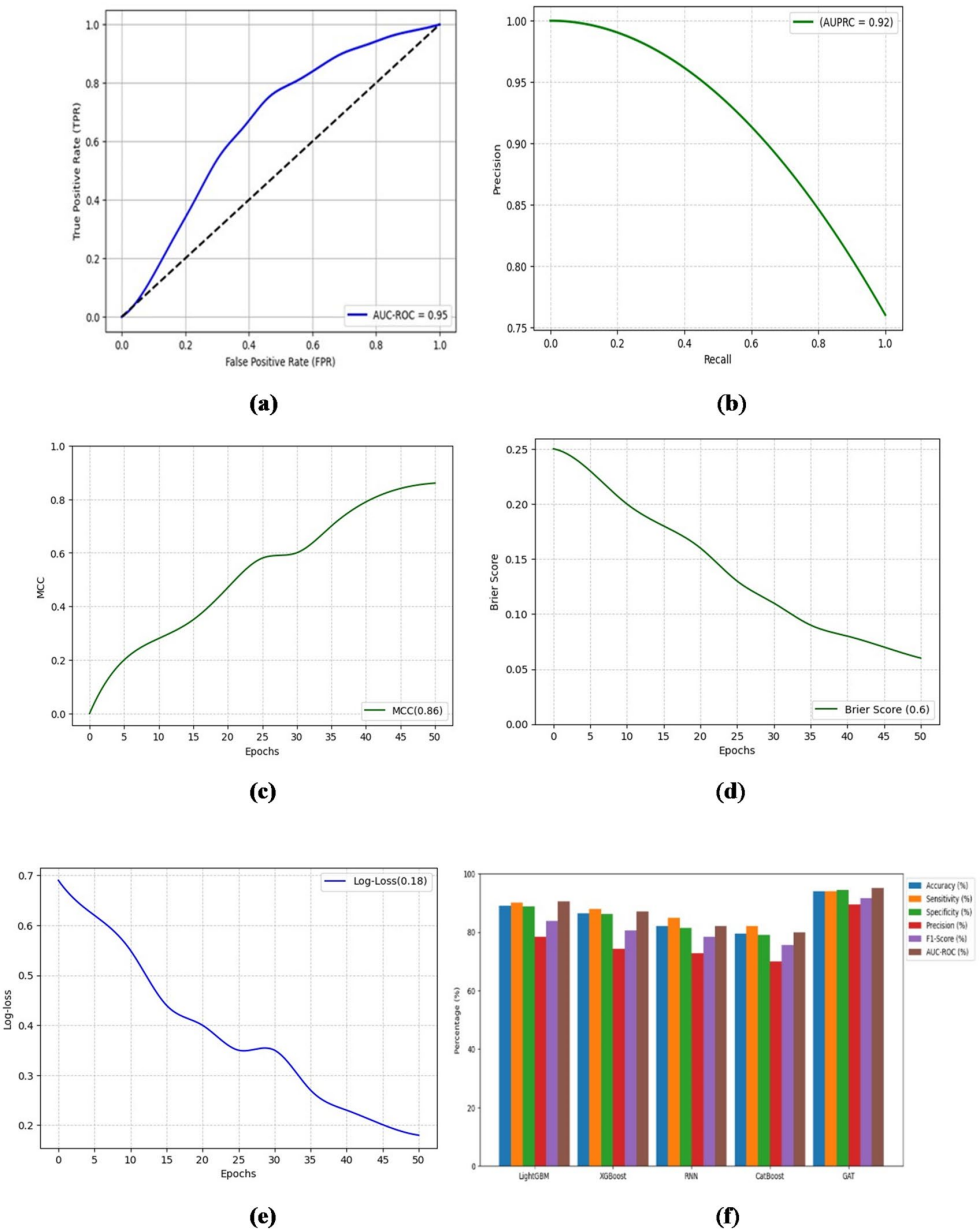
GAT model performances and comparison. (**a**) AUC-ROC. (**b**) AUPRC. (**c**) MCC. (**d**) Brier score. (**e**) Log loss. (**f**) GAT comparison on existing studies.

Centralized GAT correctly predicts 846 true positives that demonstrate its ability to foresee the onset of AKI with high precision. Figure 6a shows the AUC-ROC curve of the GAT model that evaluates their discriminative power to identify the risk of AKI in the prediction window, Fig. 6b shows the AUPRC, a measure for the precision-recall trade-off, the classification consistency is evaluated in Fig. 6c using MCC, a robust metric for imbalanced datasets, brier Score evaluates the calibration quality by comparing the reliability of prediction which is shown in Fig. 6d and e provides Log Loss, which measures predictive uncertainty, and Fig. 6f shows the comparison of the proposed GAT model to the existing models on the same dataset.

Centralized GAT achieves an AUC-ROC of 0.95, which accurately identifies important relationships between features for early AKI risk estimation. With AUPRCs of 0.92 and 0.91, respectively, the GAT model achieves a strong precision-recall balance, which confirms their ability to deliver reliable AKI risk estimation. With 0.86 of MCC, the proposed GAT provides a well-balanced classification performance. Centralized GAT has a Brier Score of 0.06, which indicates high-calibrated accuracy. The achieved Log Loss value of Centralized GAT is 0.18 in 50 epochs, thus illustrating optimal probability estimations. An ablation study presented in Table 1 utilizes the 5-fold cross-validation and was tested with alternative head counts on the dataset to validate the 8 Attention heads.

**Table 1:** Ablation study on GAT attention heads.

Number of heads	Accuracy (%)	AUC-ROC	Training time (min)
1	86	0.81	15
4	91.3	0.92	18
8	94.1	0.95	20
12	94.2	0.955	26

With one head, accuracy dropped to 86% and AUC-ROC to 0.81, as the model was less capable of modeling feature interactions. With four heads, the performance improved to 91.3% accuracy and 0.92 AUC-ROC, but was still not optimal. Twelve heads provided a marginal increase of 94.2% accuracy and 0.955 AUC-ROC but showed 20% longer training time than eight heads, confirming eight heads as optimal in terms of feature diversity and computational efficacy.

### GL-AA-GAT performance

**Fig. 7:**
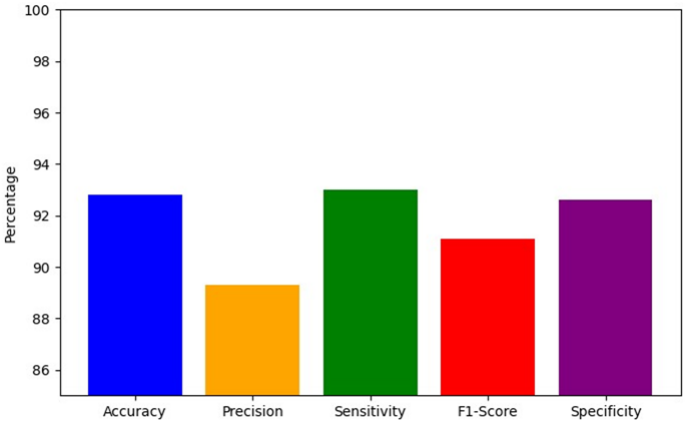
GL-AA-GAT model performance metrics for early AKI prediction.

The evaluation metrics of the decentralized GL-AA-GAT approach are shown in Fig. 7.

**Fig. 8:**
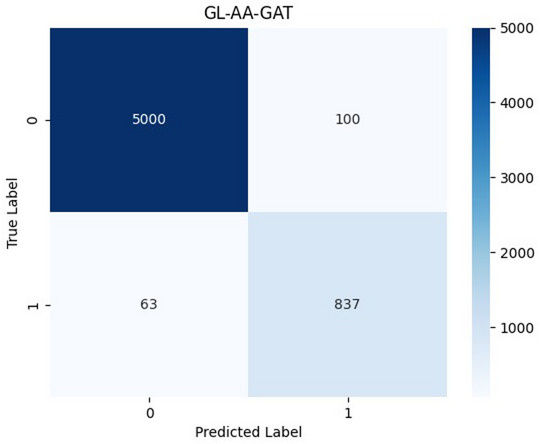
GL-AA-GAT model confusion matrix.

Results show that GL-AA-GAT achieves 92.8% accuracy while preserving privacy, which demonstrates that decentralized learning can achieve strong predictive power in multi-hospital settings. Figure 8 illustrates the confusion matrix that highlights the classification performance of the GL-AA-GAT model.

**Fig. 9:**
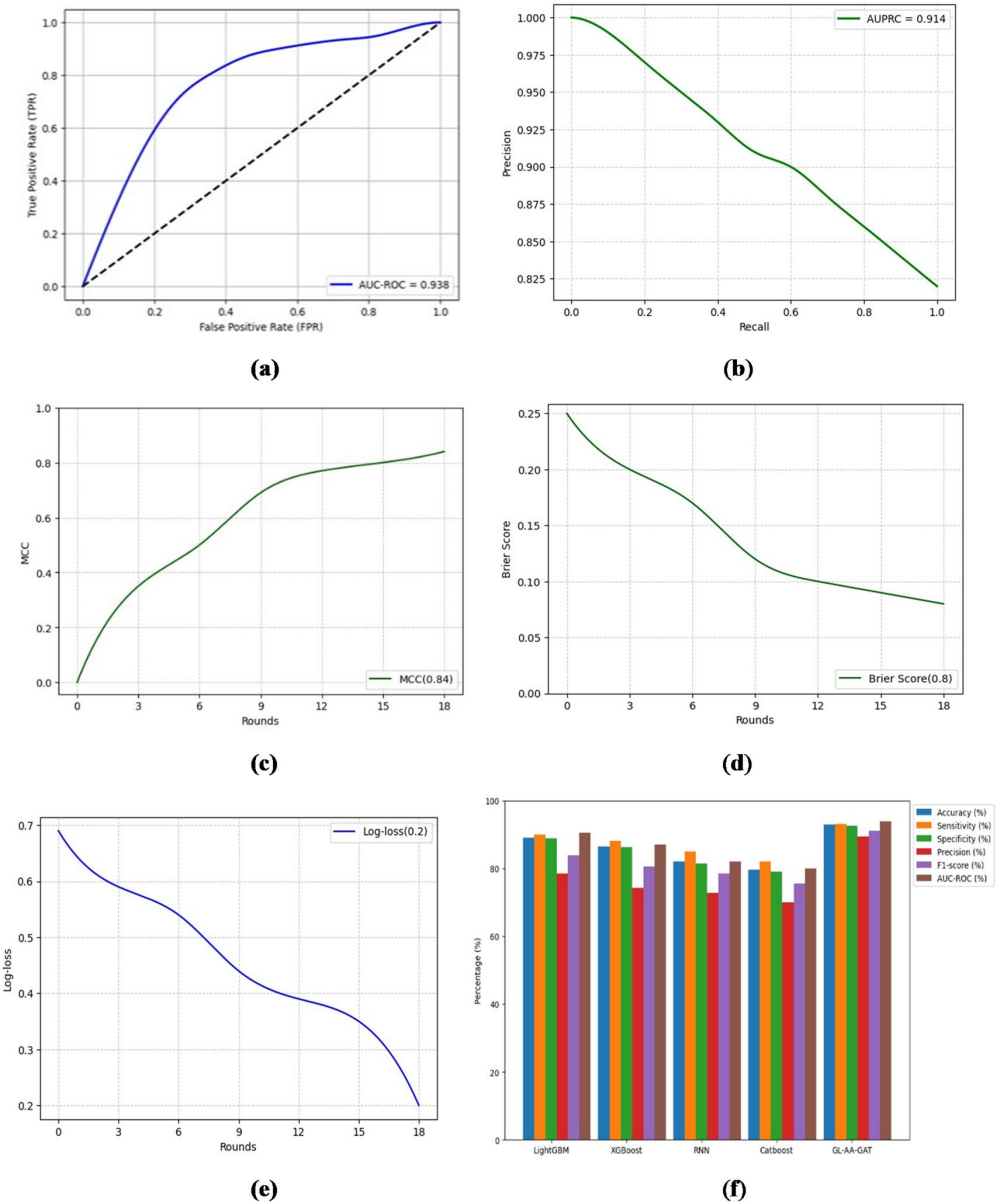
GL-AA-GAT model performances and comparison. (**a**) AUC-ROC. (**b**) AUPRC. (**c**) MCC. (**d**) Brier score. (**e**) Log loss. (**f**) GL-AA-GAT with existing studies.

GL-AA-GAT yields reliable classification performance while running in a decentralized setting and achieves 837 true positives. Figure 9a shows the GL-AAGAT model’s AUC-ROC curve to identify the risk of AKI in the prediction window, Fig. 9b shows the AUPRC, Fig. 9c displays evaluation of classification consistency using MCC, in Fig. 9d, the Brier score evaluates the calibration quality by comparing the reliability of prediction, the Log Loss shown in Fig. 9e measures predictive uncertainty, and the comparison of the proposed GL-AA-GAT approach to the existing models on the same sepsis data is displayed in Fig. 9f.

GL-AA-GAT achieves 0.938 AUC-ROC, which provides a relatively stable performance. These results demonstrate that GL-AA-GAT continues to have good predictive ability while incorporating privacy-friendly features. GL-AA-GAT model achieves a strong precision-recall balance, with AUPRC of 0.92 and 0.91, respectively, which confirms their ability to deliver reliable AKI risk estimation. GL-AA-GAT achieves 0.84 MCC, which is similar in effectiveness while improving privacy and being adaptive to the environment. For GL-AA-GAT, the calibration is very stable at a 0.08 Brier score, which reflects the reliable confidence estimations when upholding local learning. With 0.2 in 18 rounds of Log Loss, the GL-AA-GAT is still efficient in decentralized learning environments. The proposed two independent approaches in Fig. 6f, Centralized GAT, and Fig. 9f GL-AA-GAT outperform the existing methods, such as CatBoost^9^, XGBoost^7^, RNN^13^, and LightGBM^10^ by achieving high accuracy, precision, sensitivity, F1-score, specificity, and AUC-ROCs that demonstrate superior detection across different clinical settings. To put the proposed models in perspective with the existing ICU tools, the current study compared modified SOFA and partial SAPS II scores on the same data set. Table 2 shows the comparison of conventional methods of clinical scoring and proposed graph-based models to predict early AKI.

**Table 2:** Performance comparison of traditional clinical scoring systems for early AKI prediction.

Model	AUROC	AUPRC	Sensitivity	Specificity
Modified SOFA	0.66	0.42	0.62	0.65
Partial SAPS II	0.68	0.49	0.64	0.66
Centralized GAT	0.941	0.91	0.94	0.945
GL-AA-GAT	0.928	0.90	0.93	0.937

In line with previous ICU AKI studies^38,39^, these scores had moderate discriminatory power, with 0.66 (SOFA) and 0.68 (SAPS II) of the AUROC, and 0.42 and 0.49 of the AUPRC, respectively. The sensitivity and specificity were about 0.62 to 0.66. Conversely, the centralized GAT had a higher AUROC and AUPRC, whereas the decentralized GL-AA-GAT had a lower AUROC and AUPRC; both scoring systems greatly exceeded the clinical scoring systems. In Table 3, the Centralized GAT Approach, and Table 4, the GL-AA-GAT Approach (A) show the Chi-Square test that classifies accuracy differences, (B) presents the outcome of DeLong’s test for AUC-ROC to rank AKI risk, and (C) shows Hosmer-Lemeshow, which evaluates the AKI probability predictions that assess calibration on the proposed approaches.

**Table 3:** Centralized GAT approach.

Comparison	Chi-Square	*p*-value	Result
(A) Chi-Square test			
GAT vs. LightGBM	452.53	< 0.001	GAT > LightGBM
GAT vs. XGBoost	627.93	< 0.001	GAT > XGBoost
GAT vs. RNN	919.06	< 0.001	GAT > RNN
GAT vs. CatBoost	1068.77	< 0.001	GAT > CatBoost

Comparison	z-statistic	*p*-value	Result
(B) DeLong’s test			
GAT vs. LightGBM	2.12	0.034	GAT > LightGBM
GAT vs. XGBoost	3.77	< 0.001	GAT > XGBoost
GAT vs. RNN	6.13	< 0.001	GAT > RNN
GAT vs. CatBoost	7.08	< 0.001	GAT > CatBoost
GAT	6.8	0.73	

Model	Chi-Square	*p*-value	Result
(C) Hosmer-Lemeshow Test			
GAT	6.8	0.73	

It is confirmed by Chi-Square that GAT’s centralized strategy is significantly better than existing models in classification accuracy, which offers improved early AKI prediction over existing methods. This further affirms GAT’s discriminative strength, as DeLong’s test shows GAT’s centralized graph attention significantly outperforms all of the existing models in AKI risk ranking.

**Table 4:** Decentralized GL-AA-GAT approach.

Comparison	Chi-Square	*p*-value	Result
(A) Chi-Square test			
GL-AA-GAT vs. LightGBM	445.53	< 0.001	GL-AA-GAT > LightGBM
GL-AA-GAT vs. XGBoost	618.41	< 0.001	GL-AA-GAT > XGBoost
GL-AA-GAT vs. RNN	906.09	< 0.001	GL-AA-GAT > RNN
GL-AA-GAT vs. CatBoost	1054.1	< 0.001	GL-AA-GAT > CatBoost

Comparison	z-statistic	*p*-value	Result
(B) DeLong’s test			
GL-AA-GAT vs. LightGBM	1.56	0.119	GL-AA-GAT > LightGBM
GL-AA-GAT vs. XGBoost	3.21	0.001	GL-AA-GAT > XGBoost
GL-AA-GAT vs. RNN	5.57	< 0.001	GL-AA-GAT > RNN
GL-AA-GAT vs. CatBoost	6.51	< 0.001	GL-AA-GAT > CatBoost

Model	Chi-Square	*p*-value	
(C) Hosmer-Lemeshow Test			
GL-AA-GAT	7.2	0.68	

The chi-square confirms that the GL-AA-GAT indicates a significant improvement over all existing models and authenticates its applicability for privacy-preserving AKI prediction. DeLong’s test indicates that GL-AA-GAT shows good AKI risk significance, beating almost all existing models and a little margin, as in LightGBM, while being compatible with privacy under decentralized setups. The proposed two independent approaches, GAT and GL-AA-GAT, produce well-calibrated AKI probability predictions that are clinically reliable in centralized and decentralized privacy-preserving conditions. The impact of Task Scheduling on GL-AA-GAT Convergence is shown in Table 5.

**Table 5:** GL-AA-GAT task scheduling ablation.

Method	Rounds to convergence	Accuracy
Without scheduling	18	92.8
With scheduling	16	92.9

The table compares GL-AA-GAT’s convergence rounds with and without Task Scheduling. The scheduling reduces rounds by 11% maintaining accuracy. An ablation experiment presented in Table 6 shows the comparison of equal-weight aggregation to adaptive aggregation.

**Table 6:** Ablation study on GL-AA-GAT adaptive aggregation.

Configuration	Accuracy (%)	AUC-ROC	Rounds
Without adaptive aggregation	90.5	0.912	18
With adaptive aggregation	92.8	0.938	18

**Fig. 10:**
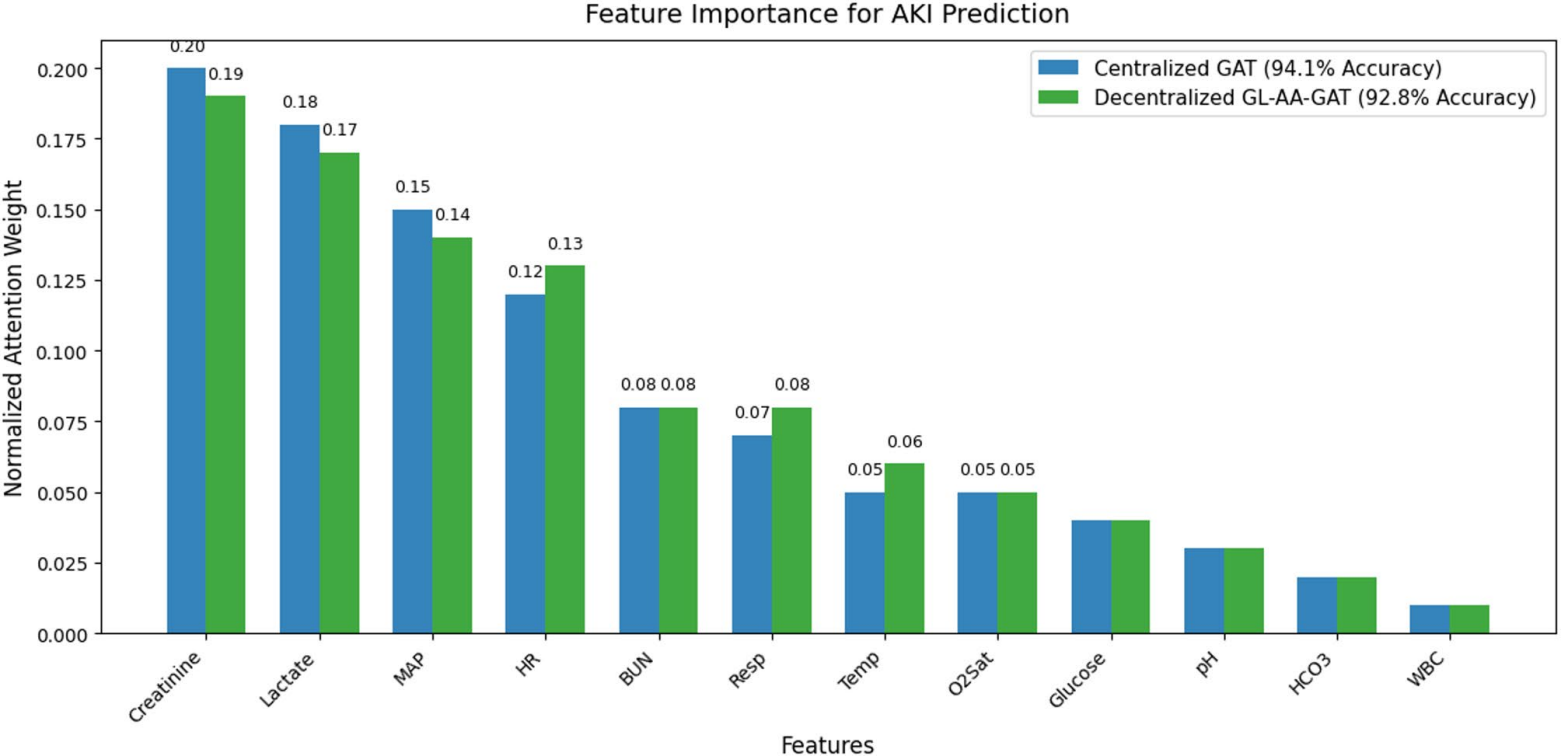
Feature importance for early AKI prediction.

The equal-weight aggregation resulted in the reduction of accuracy to 90.5% and AUC-ROC to 0.912 since node weighting was less effective. Adaptive aggregation performance is boosted by prioritizing credible node updates. Figure 10 shows a Feature importance plot for early AKI prediction.

**Fig. 11:**
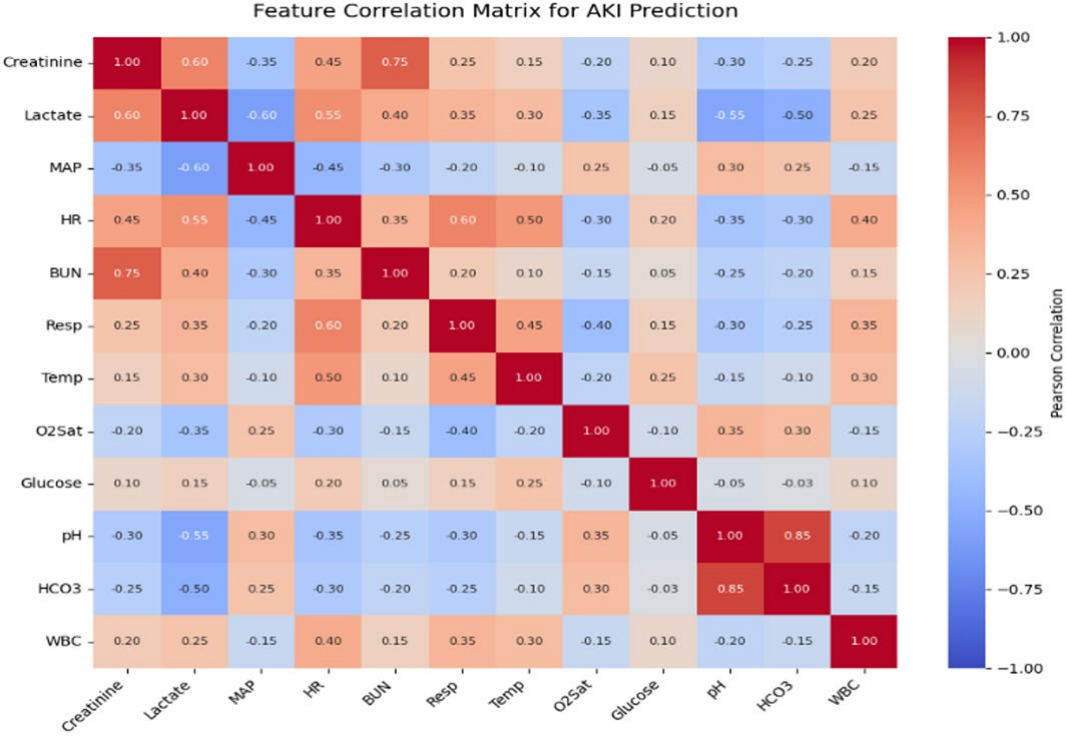
Correlation matrix. Heatmap generated using Python 3.10 with seaborn (version 0.12.2) and matplotlib (version 3.7.1). Software URLs: https://seaborn.pydata.org/ and https://matplotlib.org/.

The normalized attention weights of the 12 features are averaged across patients for Centralized GAT and Decentralized GL-AA-GAT, to show how much each contributes to early AKI detection 6–12 h earlier. To separate the effect of the graph topology, this study trained an ablation model in which features of nodes were computed alone without edges, which is equivalent to an MLP over concatenated features. This no-graph model had AUROC 0.822 and AUPRC 0.335, which is significantly lower than that of GAT. Further evaluated a fully connected adjacency, with equal weights, and also this achieved an AUROC of 0.846 and AUPRC 0.368, lower than the correlation-based graphs. These findings validate that the acquired graph structure is a significant predictive improvement over feature independence or equal strength connectivity, confirming the value of learned topology. Figure 11 displays a correlation map for early AKI prediction.

Pearson correlations among 12 features used in graph construction are averaged across patients (GAT) and post-aggregation (GL-AA-GAT), showing the physiological interactions of AKI. The results indicate strong discriminatory performance, clinical interpretability, and adaptability. Table 7 shows the Sensitivity analysis, where the values show the final-test performance at model convergence for each configuration.

**Table 7:** Sensitivity analysis.

Model	Setting	AUROC	AUPRC
Centralized GAT	*r* = 0.25, 48 h	0.949	0.915
Centralized GAT	*r* = 0.30, 48 h (baseline)	**0.95**	**0.92**
Centralized GAT	*r* = 0.35, 48 h	0.948	0.913
Centralized GAT	24 h window, *r* = 0.30	0.947	0.914
Centralized GAT	72 h window, *r* = 0.30	0.949	0.916
GL-AA-GAT	*r* = 0.25, 48 h	0.935	0.905
GL-AA-GAT	*r* = 0.30, 48 h (baseline)	**0.938**	**0.91**
GL-AA-GAT	*r* = 0.35, 48 h	0.936	0.907
GL-AA-GAT	24 h window, *r* = 0.30	0.934	0.905
GL-AA-GAT	72 h window, *r* = 0.30	0.937	0.909

To study how robust the construction of the graph and the choice of the horizon are, we conducted a sensitivity analysis using Pearson correlation thresholds (*r* = 0.25, 0.30, and 0.35) and the length of prediction (24 h, 48 h, 72 h). The outcomes are presented in Table 7, where the findings indicate that there are minimal fluctuations of predictive performance across the thresholds and windows, demonstrating the predictive performances to be stable.

This experiment further changed the number of historical time-steps of a node feature. Utilizing two steps minimized AUROC and AUPRC to 0.861 and 0.401, whereas four steps yielded 0.876 and 0.421. The 3-step setting provided the most appropriate balance, as 0.881 and 0.427, which justified the selection of the study’s design and validated the robustness to window length.

Furthermore, this study assessed the clinical utility of the proposed models using Decision Curve Analysis (DCA), pioneered by^40^. Net benefit was computed across clinically relevant threshold probabilities (0.1–0.6) using:

15 $$\:Net\:Benefit=\frac{TP}{N}-\frac{FP}{N}\times\:\frac{{p}_{t}}{1-{p}_{t}}$$

TP, FP as true and false positive, N as sample size, $\:{p}_{t}$ is the threshold. Models were compared with one another and with treat-all and treat-none strategies. Net benefit is presented in Table 8.

**Table 8:** Net benefit across threshold probabilities.

Threshold probability	Centralized GAT	GL-AA-GAT	Modified SOFA	Partial SAPS II	Treat-All	Treat-none
0.2–0.3	0.18	0.16	0.04	0.05	0.08	0.00
0.4–0.5	0.15	0.14	0.02	0.03	0.06	0.00
0.6	0.11	0.10	0.00	0.01	0.04	0.00

DCA revealed that both graph-based models showed significantly higher net benefits than traditional scoring systems and default strategies. The centralized GAT had the most clinical utility (net benefit 0.11 to 0.18) across thresholds of 0.2 to 0.6, closely followed by GL-AA-GAT (0.10 to 0.16). On the contrary, modified SOFA and partial SAPS II had minimal net benefit, often coinciding with the treat-none approach. These results prove that the suggested models are statistically better and clinically beneficial to predict the risk of AKI at the early stages. This study tested the curated 10k MIMIC III^41^ dataset from Kaggle and the updated MIMIC IV^42^ dataset from Kaggle using the proposed models for external evaluation. Using the same pipeline with required adaptations, like mapping the relatable features of physiology, for example, matching creatinine, BP, and vital signs, assigning specific values to missing each dataset, standardizing units across datasets, and normalizing based on the cohort-specific distributions to confirm the input compatibility of the model. Table 9 displays the performance of the Kaggle sepsis^31^, MIMIC III^41^, and MIMIC IV^42^ datasets.

**Table 9:** External validation on MIMIC III and MIMIC IV.

Model	Dataset	Accuracy (%)	AUROC (%)	AUPRC (%)	Sensitivity (%)
GAT	Sepsis	0.941	0.95	0.91	0.94
GAT	MIMIC III	0.892	0.856	0.835	0.85
GAT	MIMIC IV	0.917	0.883	0.853	0.879
GL-AA-GAT	Sepsis	0.928	0.938	0.90	0.93
GL-AA-GAT	MIMIC III	0.902	0.862	0.84	0.845
GL-AA-GAT	MIMIC IV	0.92	0.891	0.866	0.88

These findings place these models in a stable performance and prove the relevance of this study’s approach in a wide range of clinical cohorts and data sets. To offer strong benchmarking and comparative analysis of the models, we deployed the state-of-the-art transformer-based architectures that are suggested in the recent studies, namely: Hi-BEHRT^43^, AKI-BERT^44^, and TimelyGPT^45^. They were tested on the same dataset used in this study under the same conditions as the experiments of the proposed graph-based methods and reported in Table 10.

**Table 10:** Kaggle sepsis dataset comparison on SOTA transformer baselines.

Model	Accuracy (%)	AUROC (%)	AUPRC (%)	Sensitivity (%)
Hi-BEHRT^43^	0.927	0.91	0.86	0.91
AKI-BERT^44^	0.92	0.914	0.88	0.911
TimelyGPT^45^	0.91	0.902	0.848	0.90
GAT	0.941	0.95	0.91	0.94
GL-AA-GAT	0.928	0.938	0.90	0.93

Transformer models, as illustrated, performed well with published benchmarks. Nevertheless, our GAT and GL-AA-GAT models showed better all-important measures under identical data, experiment, and evaluation conditions, which proves the empirical merits and clinical significance of the innovative approach.

### Discussion

AKI prediction requires consideration of key biomarkers to facilitate comprehensible risk estimates from the models. Centralized graph attention methods, with very high precision, are best suited for organized medical institute settings where early intervention is crucial. Additionally, the decentralized GL-AA-GAT similarly attains high accuracy that performs best in multi-institutional settings with privacy-preservation gossip learning that maintains performance while protecting patient data. In comparison with federated learning, where the aggregation process is centralized and usually restricted by communication overhead and non-IID sensitivity^20^, gossip learning is a fully decentralized and serverless solution^25^. This design minimizes point-of-failure, increases scalability among the institutions, and supports data governance requirements without decreasing the competitive performance. Moreover, gossip learning has been demonstrated to attain the same level of convergence as federated learning in both stability and training rounds^25^, and in our experiments, GL-AA-GAT also demonstrated the same round-smooth convergence behavior. This study trained a FedAvg baseline approach on all 5-node setups for direct comparison. By converging 65 rounds, it achieved an AUROC of 0.873 and an AUPRC of 0.412; it performed slightly below the GL-AA-GAT model. All five nodes were required for each round to upload models weighing about 27 MB, which is approximately 135 MB each round and 9.1 GB overall. By comparison, the Gossip learning used about 1.8 GB in total. Gossip also tolerated node dropout without stalling, unlike FL. These results match studies^20,25^, showing gossip learning’s efficiency and reliability.

The model is simplified through clinical positioning. The technique allows the devices to communicate with each other directly, rather than passing through a central server, which reduces the delay in communication and removes bottlenecks. The GL-AA-GAT model has the number of parameters approximated to 1.2 M, which occupies around 27 MB in FP32. During training, a 3.8 GB GPU with a 128 batch size is used, and it works with an 8 to 12 GB GPU that is widely used in hospitals. It is fast and light, processing data in approximately 6 ms/round and consuming less than 150 MB of memory, which renders it appropriate for real-time applications in hospitals and clinics. Each gossip message transmits a data packet with a weight of approximately 27 MB, which means that 1.8 GB is sent in 65 rounds, compared to 9.1 GB sent during federated learning. This shows that it is applicable in normal hospital servers and that it might not need extra resources. The per-layer cost is proportional to $\:O(E\bullet\:d\bullet\:H)$ as seen in the Methodology section, communication per round scales with the number of parameters, about 27 MB, which is sufficient to verify that it is computationally feasible, as well as to explain the practical deployment outcomes observed in this paper.

This study uses publicly available, de-identified data, but in the real world, deployment has to be in accordance with data governance laws, including GDPR in Europe and HIPAA in the United States. The gossip-based approach does not require the sharing of raw patient records and, therefore, is compatible with these frameworks to facilitate privacy-sensitive predictive analytics. Practically, implementation would adhere to institutional ethical authorizations and governance guidelines, whereby the sharing and updates of models will be in line with set GDPR/HIPAA guidelines. The ethical committee controls and specific data utilization regulations can stabilize the security. The key strength of interpretability is the graph attention mechanism’s capacity to wisely prioritize vital physiological interactions between patient groups. This promotes clinician trust and usability by giving clear decision paths instead of black-box forecasts. In addition, higher AUPRC scores confirm the effectiveness of the models in dealing with imbalanced AKI prevalence, ensuring some high-risk patients are identified appropriately while minimizing unnecessary interventions. Such gains were especially noticeable compared to conventional ICU scoring systems like SOFA and SAPS II in Table 2, which had a relatively small predictive ability as per other earlier studies of ICU AKI^38,39^. Furthermore, the DCA reinforced results demonstrated that both the graph-based models provided better clinical usage over traditional systems and approaches.

Table 7 has robustness checks that ensure that the model performance remains consistent with moderate design variations. Varying the Pearson correlation threshold between 0.25 and 0.35 generated an AUROC change of $\:\le\:$ 0.002 in both models and an AUPRC change within $\:\approx\:$ 0.006, whereas varying the prediction window between 24 h and 72 h generated an AUROC change of $\:\le\:$ 0.003. These trivial effects prove our findings are not delicate to little, plausible variations in the constructions of graphs and the choice of horizon, and so prove the reproducibility of the results. We also tested attention-head counts (4, 8, 12) and monitored the change in the AUROC, lower than 0.004, showing that the setting of the attention mechanisms does not significantly influence final discrimination.

The Centralized GAT model achieves a log loss of 0.18 after 50 epochs of training without any instability, showing convergence and stability. GL-AA-GAT achieves 0.2 in 18 gossip training rounds to be stable, which is highly resilient to any node-specific variations under decentralized conditions. GL-AA-GAT adapts based on aggressive aggregation of aggregation errors and improves the stability of the model without explicit synchronization of data. Both models’ MCC progression validates their reliability on various patient samples and robust performance in different healthcare environments. The graph-based structure in both models also allows an understanding of the complex physiological dependencies in an explainable manner, unlike traditional methods.

This study demonstrates high accuracy and interpretability using features such as Creatinine, SBP, DBP, MAP, HR, Temp, Resp, Lactate, BUN, O2Sat, pH, and WBC that capture AKI risk factors in a sepsis cohort in Figs. 10 and 11, and concludes on the usefulness of these features in hospitals. Although generalizability to non-sepsis AKI could be improved by longitudinal data, this robust set al.lows for better reliability. Moreover, both SOFA and SAPS II comparisons in this paper relied on modified/partial implementations because of data limitations (e.g., no GCS, no urine output, and no comorbidity data). Even though it is in line with previous secondary analysis^38,39^, future studies with more comprehensive EHR data will be able to compare head-to-head with the entire scoring systems.

Several previous studies indicate that AKI develops in various clinical settings beyond sepsis cohorts. For example^12^, observed AKI as a common side effect of cardiac surgery, and some predictors included perioperative hemodynamic monitoring and creatinine dynamics^10^. investigated AKI among patients with heart failure, and fluid balance measurements and kidney biomarkers were essential^15^. examined the AKI among mixed populations of ICUs with a focus on longitudinal laboratory markers of AKI (serum creatinine and electrolyte variability). These studies highlight that AKI is a multifactorial disorder that is expressed in different groups of patients with varying risk factors. Even though our present assessment relied on the sepsis cohort, the suggested graph-based model is not necessarily limited to this setting. This study has validated the proposed graph-based models on the Kaggle MIMIC-III and MIMIC-IV datasets and found that the proposed models continue to be highly predictive even after the initial training cohort. This further supports evidence of model generalizability, which in turn supports the clinical applicability of the methodology in different ICU populations.

Adaptive aggregation makes the privacy and scalability of GL-AA-GAT justified despite its computational overhead. Further improvement of model scalability in predictiveness-preservation trade-offs is achieved via refined aggregation dynamics and adaptive adjustments with task scheduling by reducing gossip rounds from 18 to 16, which boosts GL-AA-GAT’s efficiency and scalability in decentralized healthcare applications. Lastly, the strength of our parameterization was also validated by consistency with previous results: the KDIGO-based 48-hour horizon and the correlation thresholds in the 0.25–0.35 range have been repeatedly tested to be predictively stable^13,33,36^. This compliance with the clinical guidelines and existing empirical research makes our framework reproducible. To be complete, this study trained a baseline Transformer model (time-series encoder, two layers, four heads, hidden size 64). It had an AUROC and AUPRC of 0.857 and 0.394 on the sepsis cohort, respectively, less than GAT and GL-AA-GAT. This is in line with recent literature that Transformers are superior in very long sequences, but graph-based models make more use of inter-variable correlations in organized physiological data. Table 11 shows the Transformer-based models in healthcare prediction.

**Table 11:** Transformer-based models in healthcare prediction.

Model	Task	Key results	Limitation
Hi-BEHRT^43^	Longitudinal EHR risk prediction	AUROC up to 0.96; AUPRC $\:+$ 8%	Struggles with ultra-long sequences
ClinicalBERT-transformer^46^	Early sepsis (MIMIC-III)	AUROC $\:\approx\:$ 0.85; AUPRC $\:\approx\:$ 0.59 (12 h)	Sensitive to missing notes
TimelyGPT^45^	Long-horizon forecasting	Recall@10 $\:\approx\:$ 70.8%	In-domain only

Newer designs of Transformers have achieved state-of-the-art findings on various healthcare tasks. Hi-BEHRT reported AUROC to 0.96 and AUPRC gains to + 8% on longitudinal EHR prediction^43^; Another study reported AUROC to $\:\approx\:$ 0.85 and AUPRC to $\:\approx\:$ 0.59 at a 12-hour horizon on early sepsis prediction with multimodal Transformers^46^; and TimelyGPT reported Recall at 10 to be $\:\approx\:$ 70.8% on irregular long-horizon forecasting^45^. The robustness of Transformers in capturing long-range and multimodal reliance assures these benchmarks. This study’s Correlation-driven graph models are complementary, as they introduced the explicit, explicable physiological relationships that the Transformers don’t inherently have, so, as a result, it intensifies clinical transparency. Therefore, this method has clear strengths, but it also has its limitations. Its applicability to groups beyond the sepsis cohort, such as other AKI populations, still needs to be explored^10,12,15^. Additionally, comparisons with SOFA and SAPS II scores were limited due to missing data, which aligns with findings from previous studies^38,39^. There is a need to evaluate the Larger dataset in real-world environments in different settings to confirm its effectiveness.

## Conclusion

This study presents two approaches, namely, Centralized GAT and GL-AA-GAT, which are two graph-based predictive models of early AKI that are highly discriminative, interpretable, and scalable. Structured clinical settings can effectively perform AKI risk stratification by centralized GAT, whereas GL-AA-GAT for decentralized learning enables privacy protection without degradation of predictive strength. The two independent approaches of this study have excellent sensitivity and specificity and are well-calibrated probability estimates, and therefore practical and usable. By integrating graph attention mechanisms, model interpretability is improved with the prioritization of key biomarkers and physiological interactions to clinician trust. Through adaptive aggregation and gossip learning, the GL-AA-GAT performs robustly and is thus amenable to the scalable and privacy-aware deployment of decentralized infrastructures, which can benefit from these. Both models approach the physical dependence in complex physiological data in a way that conventional DL methods do not, and both are more interpretable and clinically relevant than conventional deep learning methods. The models are state-of-the-art in risk prediction for AKI with wide physiological features. Gossip exchange mechanisms need to be optimized by refining for computational efficiency and convergence stability, as initiated by task scheduling’s 11% reduction in rounds, for decentralized training to be more efficient. The resistance and sensitivity tests confirmed that the model remains stable with variations in correlation thresholds, prediction horizons, and attention head. The methodological strength and clinical utility of the proposed graph-based approach were further confirmed by the comparison with the ablations, traditional scores system used in the ICU, and Transformer baselines. This foundation could be refined in future work by including longitudinal trends and tuning GL-AA-GAT concerning efficiency across a class of different clinical settings. Deployment in the real world in multi-institutional environments will provide a valid assessment of real-world deployment and the hope of seamless integration with practical privacy-preserving AI solutions for AKI risk assessment.

## Data Availability

The datasets used and/or analysed during the current study are available in the Kaggle repository, https://www.kaggle.com/datasets/salikhussaini49/prediction-of-sepsis.
